# Comparing Length and Telomere Expression at Oral Precancerous and Cancerous Stages

**DOI:** 10.30699/IJP.2024.1996330.3081

**Published:** 2024-02-15

**Authors:** Fahad Mansoor Samadi, Shaista Suhail, Manjari Sonam, Mohd Kaleem Ahmad, Vijay Kumar, Shaleen Chandra, Shadab Mohammad

**Affiliations:** 1 *Department of Oral Pathology and Microbiology, King George Medical University, Lucknow, India*; 2 *Department of Oral Pathology and Microbiology, Sardar Patel PG Institute of Dental and Medical Sciences, Lucknow, India*; 3 *Department of Biochemistry, King George Medical University, Lucknow, India*; 4 *Department of Surgical Oncology, King George Medical University, Lucknow, India*; 5 *Department of Oral Maxillofacial Surgery, King George Medical University, Lucknow, India*

**Keywords:** Carcinoma, Biomarkers, DNA, Prognosis, Telomere

## Abstract

**Background & Objective:**

Telomeres consist of repetitive G-rich nucleotides located at the end of each chromosome, acting as protein binding sites. The aim of this study was to examine the differences in telomere length in blood, saliva, and tissue samples at various stages of oral precancerous and cancerous lesions.

**Methods:**

Samples of blood, tissue, and saliva were collected from patients with oral precancerous and cancerous lesions. DNA extraction was performed. Then, a TRAP assay was conducted to assess and compare the telomere length and telomerase expression.

**Results:**

The levels of telomerase activity (TA) in the DNA samples ranged from 0.19 to 6.91 (2.05+1.37) among oral squamous cell carcinoma (OSCC) patients and from 0.17 to 4.5 (0.28+4.25) among precancerous patients. A significant difference was observed in TA levels between OSCC and precancerous samples (*t*=3.9691, *P*= 0.0000).

**Conclusion:**

Assessing the telomerase activity is crucial for studying the behavior of carcinoma in the clinical setting. The augmented telomerase expression and the length of telomere contribute to OSCC progression. Hence, this study adds a diagnostic tool that can serve as a biomarker for the early detection and prognosis of OSCC.

## Introduction

Telomeres consist of G-rich nucleotides at the ends of every chromosome that repeat and act as binding sites for a wide range of proteins (1). These telomeres being specific assemblies encompass a tandem repeat sequence of particular DNA and related proteins. They have a well-defined protective function in averting genomic loss in the DNA sequence of the proliferating cells (2-4). It has been established that with each cell division and due to certain oxidative stress reactions, these telomeres shorten in length and vary with cell type and age. Somatic cell telomeres range between 6 to 12 kb in size. In such instances, censoriously short telomeres instigate an apoptotic pathway or cellular senescence resulting in the cell division termination. For that reason, telomere shortening is considered an important mechanism for tumor suppression (5-9). It has been documented that during cell division, each chromosome in normal cells loses approximately 50100 base pairs at the 5' end (10). This process acts like a biological clock, abolishing the surviving cells for longer periods to accrue mutations or aberrations (11, 12). The infinite cell divisions lead to a decreased level of heritable genes. Hence, the shortening of telomere length is an imperative sign for the initiation of cell death due to p53-dependent aging or apoptosis (13, 14). 

Erythroplakia, oral leukoplakia, and oral submucous fibrosis are some important types of premalignant lesions or precancerous conditions of the mouth. The individuals progressing with these lesions are susceptible to advancing oral squamous cell carcinoma (OSCC) (9). According to the estimated rates, 85% of the precancerous cases originate from oral leukoplakic lesions, and 95% of oral cancers comprise squamous cell carcinomas (15, 16). The common rate of malignant transformation of any dysplastic lesion ranges from 11% to 36%, contingent on the follow-up period. Studies in the past show the association of genetic risk factors with malignancy, thus suggesting that genomic modifications play a significant role in the etiology of precancerous and cancerous lesions (17, 18). The process of oral carcinogenesis is an exceedingly intricate and multifaceted trajectory comprising of genetic modifications accretion and, with the help of oncogenes, causes induction of proteins resulting in abnormal cell growth, augmented enzymatic (telomerase) activity encouraging cell proliferation and loss of proteins that can also inhibit the abnormal cell proliferation with the help of tumor suppressor genes (19, 20). 

Hence, this study was conducted to compare the variations in telomere length in blood, saliva, and tissue samples at various stages of oral precancerous and cancerous lesions.

## Material and Methods

The present research was conducted at the King George's Medical University in Lucknow, Department of Oral Pathology and Department of Biochemistry. This study did not include any cancer patients undergoing chemotherapy, radiation, or surgery. Preliminary examinations were done on the patients for any signs or symptoms of oral cancer. Blood, tissues, and saliva samples were collected from the precancerous and cancerous patients along with control samples to perform the Telomerase Repeated Amplification Protocol (TRAP) assay to study and compare the telomere length and telomerase expression in the specimens at their respective oral precancerous and cancerous stages. DNA was isolated from the samples using the kit protocol (QIAGEN KIT).

TRAP assay was performed to calculate the telomerase activity (TA) in the samples. TRAP assay for the test reactions included two steps. This study was ethically approved by the Institutional Ethics Committee, Office of Research cell, KGMU, Lucknow, UP [ECR/262/Inst/UP/2013].

**Telomerase Expression in Tissue**

For the tissue samples, the cell extract was mixed with CX (5ˈ-CCCTTACCCTTACCCTAA-3ˈ) reverse primer and diethyl pyro carbonate (DEPC) to bring the RNA primer mix final volume to 10 μL. The mix was incubated for 10 min at 65°C and then instantly chilled on ice. The telomerase in the extract synthesized telomeric DNA oligonucleotides on TS (5ˈ-AATCCGTCGAGCAGAGTT-3ˈ) primer. The supermix was made using the kit protocol. The thermal cycler was preheated to 50°C. For the initial denaturation process, the conditions of the cycles were set at 42°C for 30 min and 94°C for 15 min. The next 3 steps of denaturation, annealing, and extension steps included 36 cycles of 94°C for 30 sec, 58°C for 30 sec, and 72°C for 30 sec. Final extensions at 72°C for 5 min and 4°C for 1 min were done. Several dilutions (1:2, 1:5, 1:10, 1:25, and 1:50) of the control RNA template went through a control reaction and later were used as the internal TA comparison standard. In the subsequent stages, polymerase chain reaction (PCR) was used to amplify the new telomerase-synthesized oligonucleotides by adding reverse CX primer when deoxynucleotide triphosphates (dNTPs) were present. The final mix was kept and run in a thermocycler, which went through logarithmic cycles of PCR in three steps. The reaction products were kept at 4°C. Finally, gel electrophoresis was performed to analyze the samples for amplification. 

**Telomerase Expression in Saliva**

Saliva samples were acquired in 25 mL of sterile saline, briefly gargled (15 s), and spat into a sterile conical tube. The samples were centrifuged at 2500×g for 15 min at 4°C to pellet down the cells. The supernatant was removed and the cell pellet was resuspended in 1 mL saline. The samples were snap-frozen in the liquid nitrogen. For use, the cell pellets were dissolved in saline, centrifuged at 13,000×g for 10 min and the supernatant was divided into 2 parts and one part was heat-treated (85°C for 15 min) to be used as negative control along with each sample. The aliquots of every supernatant were added in TRAP master mix to produce a solution of 20 mM Tris-HCl (pH 8.3), 63 mM KCl, 1.5 mM MgCI_2_, 1 mM EGTA, 0.5 mM each of deoxynucleotide triphosphate (dNTPs), 0.005% Tween 20, and 0.25 µg of T4 gene 32 protein after incubation at 30°C for 1 hr. In dilutions of 1:10 and 1:100, the negative samples were run again to recover TA to avoid telomerase (or PCR) inhibitors hindering any adequate extension. To produce a final concentration of 1.5 mM MgCl_2_, 20 mM Tris-HC1 (pH 8.3), 63 mM KCI, 0.005% Tween 20, 1 mM EGTA, 0.25 mM each of deoxynucleotide triphosphate (dNTPs), and 1.5 × 10^-17^ g of internal positive control for PCR, 25 µL of master mix, 4 µCi of (**ᵞ**^32^P) dCTP, and 10 units of Taq polymerase were added to the resuspended DNA product mixture. The amplification of microsatellite repeats (D4S243) was done utilizing modified primers where each primer contained a forward or reverse D4S243 primer on the 3' end and either CX or TS primer sequences on the 5' end of each primer. It was used as an internal control. The following primer sequences were used to amplify this control amplicon: 

[5'-CCCTTACCCTTACCCTTACCCTAATCA GTCTCTCTTTCTCCTTGCA-3' and 5'-AATCCTT CGAGCAGAGTTTAGGAGCCTGTGGTCCTGTT-3']. An approximate 200bp PCR product was derived, which was gel purified, and quantitated, and in every reaction mixture, 1.5 X l0^-17^ g were included. A wax barrier separated this mixture from 100 ng of CX primer. Then, the reactions went through incubation for 1 min at 90°C. Finally, PCR was performed with the following reaction cycle conditions: A total of 35 cycles contained denaturation of 30 sec at 95°C, 30 sec of annealing at 50°C, an extension step of 1 min at 70°C, and the last extension step of 5 min at 70°C. 

**Telomerase Expression in Blood**

To check the telomerase expression in blood, a Human TE ELISA kit was used. The microplate was pre-coated with an antibody particular to TE. Serial 2-fold dilutions of the standard solution (from 0 to 10 ng/mL) were prepared. The kit sample diluent buffer was used to dilute the plasma samples to a 1:250 ratio. The samples were tested in duplicates and incubated for 90 min at 37ºC. Then, the samples were aspirated, the biotinylated detection antibody was added, and incubated at 37ºC. The antibody was aspirated after 1 hr and the wells were washed thrice using wash buffer. The next step was the addition of HRP-conjugated antibody and incubation at 37ºC for 30 min. Then, following washing the substrate was added into the wells and incubated for 15 min at room temperature. Finally, the stop solution was added, and the optical density (OD) was recorded utilizing a plate reader at 450 nm wavelength. The equation obtained from the standard curve was used to analyze the results. The acquired results were reported as ng/mg for biopsy and ng/mL for plasma.

**Gel Electrophoresis**

The PCR products were analyzed qualitatively by performing gel electrophoresis. A 12% non-denaturing polyacrylamide gel was prepared, consisting of ethidium bromide and 10X TAE (Tris-acetate ethylene diamine tetraacetic acid) buffer, where the same 10X TAE buffer was used to run the gel and resolve the products (10 μL/each). The products were run on the gel with bromophenol blue to track the sample. In the aforementioned several dilutions, 10 μL of control reaction PCR products were also resolved on the gel. A quantity of 1 μg was taken as the minimum dilution measure of the control reaction to appreciate the detectable activity. Hence, a control reaction PCR product (1 μg) was added to each run for the later comparison. The gel electrophoresis was run at 25 V and 16 mA for 2 hr. The separated products appeared in the ladder pattern. Then, the wet gel was visualized using an ultraviolet transilluminator.

**Telomere Length Analysis by PCR**

Terminal restriction fragment (TRF) Southern blot analysis was used to determine the telomere length (TL) of the isolated DNA. The extracted DNA (10 µg) was digested with 10 units of *Hinf*I restriction endonuclease overnight at 37°C. The digested product was run on 0.5% agarose gel. The gel was depurinated in 0.25 *M *HCl, denatured in 0.4 *M *NaOH/1.5 *M *NaCl, and neutralized in 0.5 *M *tris/3 *M *NaCl following electrophoresis. The DNA sample with pH 7.5 was transferred to a positively charged nylon membrane, followed by the fixing process of incubation at 120°C for 1 hr. The probe (TTAGGG)_ 4_ for telomeres was labeled with digoxigenin (DIG) by the 3’-endutilizing DIG oligonucleotide 3'-End Labelling kit. The pre-hybridization of the nylon membrane was done in 5X saline sodium citrate (SSC), 0.1% sarkosyl anionic surfactant, 2% blocking reagent, and 0.02% sodium dodecyl sulphate (SDS) at 55°C for 1 h. A pre-hybridization solution consisting of digoxigenated oligonucleotide probes was used for hybridization. The following steps involved 10 min wash in 0.1% SDS and 2X SCC at room temperature, and incubation at 42°C for 30 min in 0.1% SDS. DIG Luminescent Detection Kit was used to detect telomeric smears on chemiluminescence film and Molecular Analyst Software (Biorad) was used to scan and analyze the bands. The density peak was accounted to be the peak TRF length. The membrane stripping was done with 30 min incubation in 0.2 M NaOH and 0.1 % SDS at 39°C. After that, the digoxigenated minisatellite probe (CAC)_5 _was used to rehybridize the membrane. Hybridization was performed for 1.5 h at 42°C. The stringent washes were done for 10 min at room temperature, followed by another washing step in 4X SSC and 0.1% SDS for 30 min at 42°C.

## Results

The TA levels were found in 90% OSCC, 35% precancerous, and 5% normal oral mucosa (NOM) samples. The levels of TA varied from 0.19 to 6.91 (2.05+1.37) in OSCC, from 0.17 to 4.5 (0.28+4.25) in precancerous, and 0.21 to 1.09 (0.54+0.27) in NOM samples. The TA level considerably varied between OSCC and precancerous samples (*t*=3.9691, *P*=0.0000). The oral rinses from all the patients with OSCC possessed TA. It was detected positive in 79.0% of OSCC cases, 51% of precancerous cases, and 6.67% of normal subjects. The TA level was found in 55% of OSCC cases, 20% of precancerous cases, and 6% of NOM samples. On comparing the telomerase expression in all specimens at oral precancerous and cancerous stages, the TA level was found to be 90% in OSCC stages, 35% in precancerous stages, and 5% in NOM subjects. The TA levels varied from 0.19 to 6.91 (2.05+1.37) in OSCC samples, from 0.17 to 4.5 (0.28+4.25) in precancerous stage samples, and 0.21 to 1.09 (0.54+0.27) in NOM samples. The TA level varied significantly between the OSCC and precancerous forms (*t*=3.9691, *P*=0.0000). The TA was detected positive in 79.0% of OSCC cases, 51% of precancerous cases, and 6.67% of NOM samples. All the OSCC cases were found to possess TA. The statistical difference was consequential, with a P-value<0.001. However, the TA expression level difference between the patients in the early and the late clinical stages was not consequential (*P*>0.05), similar to the patients with and without lymph node metastasis (*P*>0.05). 

## Discussion

Telomerase activity (TA) is acknowledged as an exceptional characteristic of malignant cells. Nevertheless, this enzyme activity was previously detected in stem cells and some somatic cells and the marker specificity has been jeopardized (21-23). Notwithstanding these factors, TA goes on to be documented as a plausible therapeutic target for the malignancy. The expression of telomerase activity and the telomere length are the most pertinent aspects in the early diagnosis of carcinomas that could provide a success rate of the succeeding treatment procedures. Growth in TAy and short telomere lengths are conjectured to assist with definite signs of carcinogenesis (24, 25). It has been established in various research projects that elevated telomerase mRNA expression levels, and TA in malignancy depicted a poor prognosis for the respective patient. Authors have hypothesized that unwarranted telomere shortening, and severe telomere uncapping might induce DNA damage responses at chromosomal ends, thus familiarized as double-strand breaks. However, the integrity of DNA damage responses was in question as to whether or not malfunctioning telomeres led to the development of cancer (26). Secondly, serious telomere shortening was hypothesized to establish an initiating factor for cellular transformation with the help of genome instability, leading to carcinogenesis (27).

Various studies have suggested that telomere length alterations or TA detection can be seen in peripheral blood leukocytes or migrating cancer cells. These results may facilitate the qualification or determination of the patients having differential cancer grades or stages. Regarding telomere length as a diagnostic factor, some studies have connected telomere length changes to the cancer progression risk (28-31). The telomere length measurement is thought to be effective for the prognosis, specifically when evaluating assays based on the blood cell telomere length. These tests are efficient and non-invasive (14). 

Maruyama* et al.* (1998) attempted to estimate the correlation between telomere length and TA using mucosa samples that endure adenoma, metaplasia, and cancer of the stoma (32). They concluded that TA induction is an early event in the development of gastric cancer and is insufficient concerning adenoma to restore telomere length following further cell divisions. They claimed that although TA is insufficient at that point to fix the reduced telomeric DNA, it is still visible in the early stages of the cancer.

Through their study, Sainger *et al.* revealed a significant utility of telomerase activation and TL in diagnosing oral carcinoma cases (33). They proposed that over-expression of the telomeric repeat binding factor (TRF 2) proteins in oral tissues depicted the loss of capping function, thus, leading to the end-to-end fusion as seen in malignant cells. Hence, telomere-dependent genomic alterations resulting from protein imbalance at the telomeric end are significant in cancer cases that could facilitate recognizing novel therapy marks.

Bau *et al.* reported that the short telomere length was related to the high risk of developing oral precancerous lesions and OSCC, which was analogous to the present study findings (9). Zhu* et al.* established a significant dose-response relation between short telomerase with a high risk of head and neck cancer and gastrointestinal tumors. They suggested that telomeres have an imperative role in different cancers (34). Few other studies also established similar findings with respect to the telomerase activities in various cancers, including those of the breast, gastrointestinal tract, and oral mucosa.

Rai *et al.* proposed that TA activation is a common phenomenon in cases of OSCC (35). They exhibited considerable variation in the levels of quantified telomerase of OSCC and normal tissues. They established the important clinical practicality of telomerase activation as a useful indication for the diagnosis and its efficacy as a marker for the OSCC prognosis. According to Pal* et al.*, the method of analyzing TL attrition of the oral mucosa, eradicating the necessity of the external reference DNA, would facilitate the TL data unanimously (36). Hence, it would serve as a significant marker for defining high-risk oral precancerous groups for a further follow-up program. 

A few previous studies have suggested that extraordinary alertness is crucial for diagnosing precancerous or cancerous lesions depending on various molecular biology techniques, high-sensitivity assays like real-time PCR, and telomerase expression and activation (37). The TA has been linked to oral carcinogenesis development, including cellular proliferation. Such actions also designate that telomerase may be an explicit marker to discriminate malignant tumors from their benign counterparts. It has been well-established that telomerase expression seemed slightly higher in tumors with longer telomeres and larger, aggressive tumors. 

All in all, the expression of telomerase does not correlate with the outcome of the disease but could be camouflaged by adjuvant treatment. Hence, it could be suggested from various studies that TA could be a significant factor in defining the choice of adjuvant treatment for carcinoma patients (29, 38).

## Conclusion

Telomerase re-activation is an obligatory step of cellular immortalization that serves to be an exclusive marker of abnormal cells that might get targeted selectively. Increased telomerase expression and telomere length occur during human oral carcinogenesis, aiding in the development of mouth cancer, which could be deduced from the current study. The current research findings can be a supplementary mean and act as a biomarker to assess the oral carcinoma prognosis. As previous literature has shown that extensive analyses have been conducted for the regulation mechanisms in TA, novel methods for inhibiting TA may be recognized and adopted for cancer gene therapy. However, future clinical trials would indeed govern whether such an inhibition process is feasible in reducing the carcinoma mortality rate.
